# Development of a submicron emulsion-based delivery system to improve the anti-inflammatory activity of urolithin A

**DOI:** 10.3389/jpps.2025.15553

**Published:** 2025-11-07

**Authors:** Elisabetta Esposito, Valentyn Dzyhovskyi, Federico Santamaria, Lorenza Marvelli, Paola Boldrini, Paolo Mariani, Alessia Pepe, Maria Grazia Ortore, Mascia Benedusi, Giuseppe Valacchi, Luca Ferraro

**Affiliations:** 1 Department of Chemical, Pharmaceutical and Agricultural Sciences, University of Ferrara, Ferrara, Italy; 2 Department of Translational Medicine and for Romagna, University of Ferrara, Ferrara, Italy; 3 Laboratory for Technologies of Advanced Therapies “LTTA”-Electron Microscopy Center, University of Ferrara, Ferrara, Italy; 4 Department of Life and Environmental Sciences, Polytechnic University of Marche, Ancona, Italy; 5 Department of Neurosciences and Rehabilitation, University of Ferrara, Ferrara, Italy; 6 Animal Science Department, Plants for Human Health Institute, NC State University, Kannapolis, NC, United States; 7 Department of Food and Nutrition, Kyung Hee University, Seoul, Republic of Korea; 8 Department of Environmental Science and Prevention, University of Ferrara, Ferrara, Italy; 9 Department of Life Sciences and Biotechnology, University of Ferrara, Ferrara, Italy

**Keywords:** urolithin-A, ellagic acid, submicron emulsion, dialysis, carrageenan-induced paw edema model

## Abstract

**Objective:**

Despite the antioxidant, anti-inflammatory, and anti-cellular-aging activities of urolithin A (UroA), a naturally occurring postbiotic, its high lipophilicity hampers its pharmaceutical application. To overcome this limitation improving its stability and bioavailability, submicron emulsions (S-EMs) were designed.

**Methods:**

Nineteen formulations (S-EM 1/S-EM 19) were prepared by two different methodologies. S-EMs were characterized evaluating macroscopical appearance and size distribution by photon correlation spectroscopy (PCS). One selected S-EM was loaded with UroA and characterized by PCS, transmission electron microscopy (TEM), small angle x-ray scattering (SAXS) and Fourier-transform infrared spectroscopy (FT-IR). Z potential, pH and syringeability were evaluated. UroA entrapment was studied efficiency by ultrafiltration and HPLC, while *in vitro* release by dialysis. Cytotoxicity was evaluated by the 3-(4,5-dimethylthiazol-2-yl)-2,5-diphenyltetrazolium bromide) (MTT) viability test on primary dermal human fibroblasts. The anti-inflammatory activity of S-EM-UroA was evaluated at 3, 6, and 24 h post-injection using the carrageenan-induced paw edema model in male C57BL/6 mice, and compared with UroA suspension and unloaded S-EM.

**Results:**

The preformulative study enabled to select method and composition for S-EM preparation. S-EM 18 was selected for UroA loading (SEM-UroA), due to mean diameter, zeta potential, pH and syringeability suitable for intraperitoneal administration. The loading of UroA (0.2 mg/mL) did not influence S-EM physicochemical features, while maintaining technological properties for 3 months. *In vitro* drug release showed a biphasic profile, 2.35-fold faster in the case of SEM-UroA compared to the drug suspension. *In vitro* studies revealed absence of cytotoxicity at concentrations up to 5 µM. *In vivo* studies, conducted as a first step in assessing the potential of S-EM-UroA, demonstrated a dose-dependent anti-inflammatory effect. Specifically, S-EM-UroA at 2 mg/kg reduced paw edema at 24 h (*p* < 00.5; One-Way ANOVA followed by Tukey’s test), and at 4 mg/kg significantly reduced edema at all time points (*p* < 0.01), whereas the UroA suspension or S-EM had no effect on carrageenan-induced paw edema at any time point.

**Conclusion:**

These findings underscore the potential of UroA loaded S-EM as an effective delivery system, demonstrating its superiority over simple UroA suspensions in enhancing the systemic anti-inflammatory effects of the postbiotic.

## Introduction

Urolithin A (UroA) is part of the urolithin family, products of the intestinal bacterial metabolism of ellagic acid, which is widely present in fruits such as pomegranates, berries, walnuts, and in some medicinal plants like sea buckthorn [1]. UroA has gained significant attention in recent years due to its diverse and promising pharmaceutical properties. Unlike many bioactive compounds directly consumed in the diet, UroA is not found in foods themselves, being instead a metabolite produced in the human gut from the enzymatic transformation of dietary ellagitannins and ellagic acid [1–3]. The UroA origin and production are variable, depending on the intricate interplay between diet and the host gut microbiota [4]. UroA has a plethora of biological activities, primarily recognized for its role in promoting mitophagy, a crucial cellular process for clearing damaged mitochondria, thereby enhancing cellular health, and combating age-related decline [5, 6]. Beyond this, UroA exhibits potent antioxidant and anti-inflammatory effects, that contribute to its potential in mitigating oxidative stress and chronic inflammation, significant factors in many chronic diseases [7]. Because of its diverse effects, UroA shows promise for treating numerous health conditions, including muscle health, neuroprotection, and metabolic regulation, highlighting its significance for ongoing scientific investigation [2]. Both preclinical [8] and human studies [6] have demonstrated the safety of orally administered UroA, leading the FDA to grant it Generally Recognized as Safe (GRAS) status for use in food products [9].

In recent years, several studies have suggested that UroA could be partly responsible for the benefits against neurodegenerative diseases, such as Alzheimer’s or Parkinson’s, attributed to the consumption of ellagitannin-rich foods [7, 10, 11]. In this regard, some research groups are conducting studies on neuronal lesions and/or neurodegenerative diseases through animal models. These investigations indicate that the oral and intraperitoneal administration of UroA can improve cognitive impairment, brain aging, and can reduce the accumulation of amyloid plaques [3, 12, 13].

The beneficial effects of UroA on brain function during aging are associated with: (i) the reduction of inflammation and oxidative stress, which play an important role in the pathogenesis of many chronic diseases, such as cardiovascular, metabolic, neurodegenerative diseases, cancer and in the aging process [12, 14, 15]; (ii) the promotion of mitophagy (leading to the selective degradation of damaged or malfunctioning mitochondria) and the maintenance of normal mitochondrial function [16]; (iii) the inhibition of beta-amyloid peptide and tau protein accumulation, and the regulation of tryptophan metabolism [12, 17].

However, despite the evidence that UroA can activate signaling pathways against cellular aging, the direct or primary targets of UroA have not yet been fully clarified [12].

Notwithstanding its effectiveness, UroA low solubility has limited its use in clinical settings. Indeed, UroA is characterized by a poor solubility in aqueous buffers, being instead soluble in unphysiological organic solvents, such as dimethyl sulfoxide and dimethyl formamide [18]. In this respect, specific formulative approaches are required to obtain a biocompatible delivery system for UroA, thus avoiding organic solvents to assure safety, while maximizing its therapeutic potential [19]. Recently, different studies have been published concerning specialized drug delivery systems for UroA administration, spanning from nanoparticles to liposomes [20–23].

Submicron-emulsions (S-EM) represent a significant advancement in pharmaceutical formulation, offering a versatile platform for drug delivery [24, 25]. Defined by droplet sizes typically ranging from 10 to 500 nm, S-EM bridge the gap between conventional emulsions and nano-sized carriers, providing unique advantages for enhancing the therapeutic efficacy of various drugs [26]. Their submicron globule size contributes to an increased kinetic stability, reducing creaming and sedimentation, and to an increased interfacial area, thus improving drug solubilization and absorption [19]. S-EM are particularly valuable for drugs with poor aqueous solubility, as they effectively encapsulate lipophilic compounds, thereby enhancing their bioavailability and enabling administration via diverse routes, including oral, parenteral, ocular, and transdermal. Beyond their solubilizing power, these systems offer benefits such as reduced first-pass metabolism, potential for targeted drug delivery, and protection of sensitive active pharmaceutical ingredients from degradation, positioning S-EM as a powerful tool for developing more effective and patient-compliant dosage forms [26].

This study aims to develop a UroA formulation suitable for intraperitoneal delivery, with the goal of enabling future investigations into its therapeutic potential for neurodegenerative disorders, including Alzheimer’s disease.

A preformulative study was conducted to prepare S-EM suitable for UroA loading. Particular attention was devoted to employ excipients compatible with intraperitoneal administration. Selected S-EMs were characterized for their size distribution, morphology, zeta potential, and pH. The inner organization of S-EM was studied by small angle X-ray scattering and Fourier-transform infrared spectroscopy. UroA release kinetics were investigated *in vitro* by dialysis, while cytotoxicity was evaluated *in vitro* on fibroblasts. Finally, as a first step in assessing the *in vivo* potential advantages of the developed S-EM compared to UroA suspension in dimethyl sulfoxide (DMSO)/saline 1:10 v/v (SUSP UroA), the carrageenan-induced acute inflammatory model was used.

## Materials and methods

### Materials

Urolithin-A (3,8-dihydroxy-6H-benzo[c]chromen-6-one; UroA) produced by Tocris Bioscience (Bristol, United Kingdom) Witarix MCT (triglyceride of saturated vegetable oil-derived caprylic and capric fatty acids, oil phase, OF) from IOI Oleo GmbH (Hamburg, Germany) were employed. PEG 400 (polyethylene glycol 400), glycerol (GL), and polysorbate 80 (polyoxyethylene sorbitan monooleate, tween 80, TW80) were bought from Merck Life Science S.r.l. (Milan, Italy). Solvents were of HPLC grade, and all other chemicals were of analytical grade.

### Preparation of submicron emulsions

METHOD 1: The components of the dispersed phase (typically, to obtain 10 g of S-EM: OF 0.2 g, TW80 0.2 g, and ethanol 0.6 g) were weighed in a vial and subjected to magnetic stirring at 750 r.p.m. for 2–3 min (IKA RCT basic, IKA®-Werke GmbH and Co. KG, Staufen, Germany) to homogenize the mixture. The aqueous dispersing phase, typically composed of water 4.5 g and GL 4.5 g, was added dropwise to the dispersed phase (1:9, v/v) and magnetically stirred at 1,275 rpm for 30 min.

METHOD 2: The components of the dispersed phase (typically, to obtain 10 g of S-EM: OF 0.2 g, TW80 0.2 g, and ethanol 0.6 g) were weighed in a vial and subjected to magnetic stirring at 750 r.p.m. for 2–3 min to homogenize the mixture. The aqueous dispersing phase, typically composed of water 4.5 g and GL 4.5 g, was added dropwise to the dispersed phase (1:9, v/v) and magnetically stirred at 1,275 rpm for 30 min. Afterwards the emulsion was subjected to homogenization (ULTRA-TURRAX T 10 BASIC, IKA WERKE) at 25,000 rpm for 2 min.

In both cases the formulations were then stored in the dark at room temperature. For the formulative study A total of 38 different S-EMs were prepared in triplicate. Each formulation was prepared alternately with method 1 or method 2. Thus, 19 different compositions and two methods were tested. 15 compositions were based on OF, TW80, and EtOH, and 4 with the addition of glycerol to the aqueous phase.

One UroA-loaded S-EM was prepared adding the drug directly to the dispersed phase and mixing magnetically until complete dissolution, followed by the protocol described for method 2. The final UroA concentration was 0.2 mg/mL. The formulation was then stored in the dark at 4 °C.

### Morphological analysis by transmission electron microscopy (TEM)

Morphological analysis of S-EM was performed using TEM. The sample was negatively stained, depositing a sample drop on a grid covered with a collodion film. The excess drop was removed after 5 min from the grid with filter paper to keep a light veil of sample on the supporting substrate. A drop of 1% phosphotungstic acid was placed on the grid for 5 min and then removed with filter paper to surround the nanosystems deposited on the grid and adhere to their surface. Then the grid was observed with a Talos L120 G2 Trasmission Electron Microscope (Thermo Fisher Scientific Eindhoven-NL).

### Photon correlation spectroscopy (PCS)

The size distribution of S-EM was evaluated measuring the values of Z-Average, i.e., the mean hydrodynamic diameter, and the polydispersity indexes (PI) by PCS (Zetasizer Nano-S90, Malvern Instr., Malvern, England). The instrument is equipped with a 5 mW helium neon laser, producing a wavelength output of 633 nm. The analyses were conducted at 25 °C with a 90° detection angle and 120 s equilibration time. The samples were diluted 1:10 (v/v) with bi-distilled water. Size distribution was obtained by the “CONTIN” method. Each analysis was carried out in triplicate from which the mean was derived.

### Zeta potential

Zeta potential (Z-potential) was evaluated using Zetasizer Pro (Malvern Instr., Malvern, England). This technique involves the measurement of scattering of incident laser light by moving particles according to their electrophoretic mobility. The instrument is equipped with a 4 mW helium neon laser that produces a wavelength output of 632.28 nm. The analyses were conducted at 25 °C. The samples were diluted 1:10 (v/v) with bi-distilled water inside vials and then put (1 mL) inside cuvette DTS1070 (Malvern Instr., Malvern, England).

### Measurement of pH

The formulation pH was measured using Crison Basic C20 pH meter (Crison Instruments,S.A., Alella, Barcellona, Spain) equipped with a pH 50 12 - Hach electrode.

### Syringeability

The syringeability of S-EM was manually tested 24 h after preparation using 1-mL plastic syringe (plunger diameter 2 mm) equipped with a 26-G needle.

### Small angle X-ray scattering (SAXS)

SAXS experiments were performed at the Austro-SAXS beamline of Elettra Synchrotron (Trieste, Italy) [27]. Samples were put in a quartz capillary with a diameter of 1.5 mm, thermostated within a KPR (Peltier heating/cooling) sample holder (Anton Paar, Graz, Austria). Experiments were performed at 25 and 37 °C using exposure times of 10 s, with 18 repeated frames acquired for each sample to minimize potential radiation damage. The obtained two-dimensional (2D) data were corrected for background and detector efficiency and then radially averaged to derive the scattered intensity I(Q) as a function of the modulus of the scattering vector, Q = 4π sinθ/λ (where 2θ is the scattering angle and λ the X-ray wavelength, equal to 0.154 nm). The used sample-to-detector distance provided a Q-range between 0.1 and 5 nm^-1^. The detector used for the measurement was a 2D Pilatus3 1M Detector System with a pixel size of 172 × 172 μm^2^.

### Fourier-transform infrared spectroscopy (FT-IR)

The empty S-EMs, the UroA-loaded S-EM, and their components (GL, OF, TW80, and UroA) were analyzed by FT-IR spectroscopy. The analyses were performed by a BRUKER VERTEX 70 spectrophotometer (4000-400 cm^−1^) (Milano, Italy) using the diffuse reflectance technique on a solid KBr sample. To avoid altering the structure of the formulations under examination and maintain their hydration, the samples were prepared by directly depositing 10 µL in 100 mg of anhydrous KBr and mixing with a mortar.

### Entrapment efficiency

The amount of UroA effectively associated with the nanodroplets was evaluated by an ultrafiltration procedure. Briefly, 500 µL of S-EM were loaded into Microcon centrifugal filter devices equipped with YM-10 membranes (NMWCO 3 kDa, Sigma-Aldrich, St. Louis, MO, USA) and centrifuged in a Spectrafuge™ 24D Digital Microcentrifuge (Infitek Inc., Spokane, WA, USA) at 4000 rpm for 20 min.

The S-EM nanodroplet-retained fraction was then 10-fold diluted with ethanol, magnetically stirred for 30 min (IKA RCT basic, IKA®-Werke GmbH and Co. KG, Staufen, Germany), filtered through 0.22 μm nylon membranes (Whatman, Germany), and subsequently subjected to High-Performance Liquid Chromatography analysis (HPLC), according to the below reported method. In parallel, a reference aliquot (100 µL) of the whole S-EM was processed under the same conditions to determine the total drug content.

The entrapment efficiency (EE) was calculated according to the relationship reported in Equation 1:

$$\text{EE}=D/T_{D}\times 100$$ (1)

where D corresponds to the amount of UroA retained within the nanodroplets, and T_D_ is the total UroA content in the formulation.

### HPLC analysis

The HPLC analyses were performed using Perkin Elmer Series 200 HPLC systems (PerkinElmer, Waltham, MA, USA), equipped with a micropump, an auto sampler, and an UV detector. A stainless-steel C-18 reverse-phase column (15 × 0.46 cm) packed with 5 µm particles (Hypersil BDSC18 Thermo Fisher Scientific S.p.A., Milan, Italy) was eluted at a flow rate of 1 mL/min with a mobile phase composed of 30% acetonitrile, 70% water, and 0.1% TFA; the wavelength for UroA was 305 nm. The mobile phase flow rate was maintained at 1 mL/min, and at this flow rate, the retention time of UroA was approximately 5.5 min. The quantitative analysis was performed by constructing a calibration curve based on a solution of UroA (4 mg/mL) in acetonitrile with 10% dimethyl sulfoxide (DMSO), and subsequently diluted in acetonitrile in various ratios. The straight line obtained with the equation y = 4939.4 x + 3.2856 showed a correlation coefficient R = 0.9991.

### *In vitro* drug release study

The *in vitro* release of UroA from S-EM was determined by dialysis method. Briefly, 1 mL of S-EM UroA (0.2 mg/g) and a UroA suspension in DMSO/saline 1:10 (v/v) at the same concentration (SUSP UroA) were injected into the dialysis tube (Pur-A-Lyzer™ Maxi Dialysis Tube 0.1–3 mL, MWCO, 6–8 kDa, Merck, Milan, Italy), previously filled with 1 mL of bidistilled water. The dialysis tube was then placed into 200 mL of receiving phase constituted of 0.9% NaCl and 1% TW80 maintained under constant magnetic stirring at 37 ± 1 °C. At predetermined time intervals, 1 mL of the receiving phase was then sampled and subjected to HPLC analysis to determine the amount of active ingredient released over time. Each sample was replaced with an equal volume of receiving phase. UroA released amounts at each predetermined time were determined five times in independent experiments, calculating the mean values ± s.d. The cumulative percentage of drug released with respect to the total amount of drug loaded in the formulation was plotted against the time to obtain the release profile. Statistical comparison was evaluated by a Paired samples t-Student test considering 5 different experiments (n = 5 per group) comparing two different samples, making the paired test appropriate.

To gain insight into the release mechanism, the fitting of the experimental release data was analyzed using four different mathematical release models: zero-order kinetics (cumulative % drug released vs. time), first-order kinetics (log cumulative % drug remaining vs. time), Higuchi and Peppas (log cumulative % drug released vs. log time) models. The suitability of the model fits was verified using the DDsolver add-in for Excel 2016 (Version 2312 Build 16.0.17126.20126), using the correlation coefficient (R^2^) as indicator of the best fitting, for each of the considered models.

### Stability studies

A stability study was carried out over a period of 3 months, evaluating once a month size distribution and Z-potential of S-EM UroA stored at 4 °C and protected from light (n = 4). In addition, the chemical stability of UroA was evaluated by HPLC analysis in S-EM samples stored in the same conditions (n = 4).

### Cell viability and cytotoxicity study

The cytotoxicity study was performed on primary dermal human fibroblasts obtained from 3-mm skin biopsies taken from healthy donors [28], grown in Dulbecco’s modified Eagle’s medium (DMEM, Lonza, Milan, Italy) low glucose (1.0 g/L) supplemented with 10% foetal bovine serum (FBS, EuroClone, Milan, Italy), 1% L-glutamine (Lonza, Milan, Italy), and 1% penicillin-streptomycin (Pen-Strep, Lonza, Milan, Italy). The cells were incubated at 37 °C in 95% air and 5% CO_2_ until confluence.

To determine the viability of human fibroblasts, the MTT (3-(4,5-dimethylthiazol-2-yl)-2,5-diphenyltetrazolium bromide) cell viability assay, was used. Namely, S-EM, S-EM UroA 0.2 mg/L, SUSP UroA, and DMSO/saline (1:10 v/v) were evaluated in two independent experiments performed in triplicate. Fibroblasts were plated in 96-well plates at a density of 2 × 10^4^ cells/well in 200 μL of medium. Twenty-four h after seeding, the cells were treated with increasing concentrations from 1 to 200 μM of the above formulations for 24 h. The medium containing the treatments was then discarded, while 110 µL of 0.5 mg/mL MTT solution was added to each well. The plate was then incubated for 4 h at 37 °C in a dark incubator. The MTT solution was then removed, taking care not to remove the formazan crystals that had formed, which were dissolved by adding 100 µL of DMSO to the well. The plate was then placed at 37 °C for 15 min, in the dark. After 5 min of shaking, the absorbance of the solution was measured using a spectrophotometer at 590 nm, using 670 nm as the reference wavelength. This was then converted into a percentage of viability compared to the control cells.

Curve fitting and statistical analysis were performed using GraphPad Prism software, version 8 (GraphPad Software Inc., San Diego, CA). Data were fitted by nonlinear regression using a four-parameter logistic (4PL) model with a variable slope, constraining the bottom to values greater than 0.0 to avoid negative viability estimated on log-transformed x-axes. The statistical significance of concentration-dependent effects was assessed by one-way ANOVA, followed by Dunnett’s *post hoc* test for multiple comparisons and statistical significance was indicated by a two-tailed *p*-value 0.05. All data are presented as mean ± standard deviation (s.d.).

### *In vivo* study

The anti-inflammatory activity of SUSP UroA and S-EM UroA was compared using the carrageenan-induced paw edema model in male C57BL/6 mice (30–35 g body weight). Animals were housed under standard laboratory conditions (12:12 h light–dark cycle, 22 ± 1 °C, 50–60% relative humidity) with *ad libitum* access to food and water.

Mice were randomly assigned to groups of six and treated (intraperitoneally; i.p.) 2 h prior to carrageenan injection with one of the following: DMSO/saline (1:10 v/v), SUSP UroA 2 mg/kg, SUSP UroA 4 mg/kg, S-EM, or S-EM loaded with UroA at 2 or 4 mg/kg. Edema was induced via intraplantar injection (30 μL) of λ-carrageenan (15 mg/mL; Sigma Chemical Co.) into the right hind paw, under anesthesia (1.5% mixture of isoflurane and air). The contralateral paw received 30 μL of saline and served as the negative control, showing no measurable edema.

Paw volume was measured using a plethysmometer (Ugo Basile, Comerio, Italy) before carrageenan injection (baseline) and at 3-, 6-, and 24-h post-injection. Edema was expressed as the increase in paw volume at each time point relative to the baseline measurement.

Edema volumes were expressed as mean ± standard error of the mean (SEM) for each group (n = 6) and analyzed using one-way analysis of variance (ANOVA), followed by Tukey’s test for multiple comparisons.

All *in vivo* experiments were performed in accordance with the European Communities Council Directive of September 2010 (2010/63/EU), as verified by the Animal Welfare Committee (OPBA) of the University of Ferrara. The experimental procedures have been approved by the Italian Ministry of Health. Adequate measures were taken to minimize the number of animals used and animal pain and discomfort.

## Results

### Preformulative study of submicron emulsions (S-EM)

A preformulation study was conducted with the goal of obtaining a homogeneous disperse system constituted of biocompatible excipients suitable for UroA solubilization.

As reported in Table 1, for the disperse phase of S-EM the following components were employed: a biocompatible oil phase constituted of a blend of capric and caprylic acid triglycerides (40:60, v/v) (OF), the surfactant TW80 as an emulsifier, and EtOH as a cosurfactant and a cosolvent, to stabilize the system and improve drug solubilization [29, 30].

**TABLE 1:** Composition (%, w/w) and size distribution parameters of S-EM produced by methods (1), (2).

Formulation	OF^1^	TW80^2^	EtOH^3^	H_2_0	Method^4^	Z-AV^5^ (nm) ± s.d.	PI^6^ ± s.d.	Macroscopic Aspect
S-EM 1	1.0	5.0	4.0	90.0	1	304.8 ± 12.5	0.25 ± 0.04	homogeneous
					2	204.3 ± 10.4	0.38 ± 0.02	homogeneous
S-EM 2	1.0	4.0	5.0	90.0	1	441.6 ± 12.5	0.21 ± 0.02	homogeneous
					2	241.1 ± 7.3	0.36 ± 0.01	homogeneous
S-EM 3	1.0	3.0	6.0	90.0	1	543.5 ± 21.4	0.214 ± 0.02	creaming
					2	321.1 ± 8.5	0.32 ± 0.03	homogeneous
S-EM 4	1.0	2.0	7.0	90.0	1	734.1 ± 25.6	0.13 ± 0.12	creaming
					2	413.5 ± 30.2	0.31 ± 0.20	creaming
S-EM 5	1.0	6.0	3.0	90.0	1	176.9 ± 12.5	0.23 ± 0.03	homogeneous
					2	156.2 ± 10.3	0.30 ± 0.01	homogeneous
S-EM 6	1.0	8.0	1.0	90.0	1	13.8 ± 0.4	0.16 ± 0.02	homogeneous
					2	42.2 ± 3.4	0.10 ± 0.08	homogeneous
S-EM 7	2.0	2.0	6.0	90.0	1	484.0 ± 21.3	0.12 ± 0.13	creaming
					2	476.9 ± 30.8	0.16 ± 0.11	creaming
S-EM 8	2.0	3.0	5.0	90.0	1	427.8 ± 20.4	0.20 ± 0.15	creaming
					2	606.0 ± 30.5	0.23 ± 0.13	creaming
S-EM 9	2.0	7.0	1.0	90.0	1	130.3 ± 0.1	0.17 ± 0.05	homogeneous
					2	262.8 ± 12.5	0.39 ± 0.09	creaming
S-EM 10	2.5	5.0	2.5	90.0	1	286.4 ± 22.2	0.21 ± 0.15	creaming
					2	384.2 ± 25.3	0.22 ± 0.12	creaming
S-EM 11	3.0	2.0	5.0	90.0	1	476.4 ± 32.5	0.23 ± 0.14	creaming
					2	683.3 ± 42.4	0.01 ± 0.11	creaming
S-EM 12	3.0	6.0	1.0	90.0	1	237.1 ± 10.3	0.27 ± 0.15	creaming
					2	337.3 ± 12.5	0.25 ± 0.12	creaming
S-EM 13	3.0	7.0	—	90.0	1	150.3 ± 8.6	0.29 ± 0.04	homogeneous
					2	229.0 ± 10.3	0.43 ± 0.01	homogeneous
S-EM 14	4.0	5.0	1.0	90.0	1	329.1 ± 33.2	0.38 ± 0.23	creaming
					2	639.2 ± 43.0	0.23 ± 0.14	creaming
S-EM 15	6.5	2.5	1.0	90.0	1	243.8 ± 23.5	0.31 ± 0.24	creaming
					2	587.8 ± 35.2	0.21 ± 0.09	creaming

1: oil phase, blend of capric and caprylic acid triglycerides (40:60, v/v); 2: tween 80, polysorbate 80; 3: ethanol; 4: Method of S-EM preparation; 5: Z Average; 6: polydispersity index. s.d.: standard deviation; data are the mean of 4 independent determinations on different batches.

As a first approach, S-EMs were produced using Method (1), maintaining constant the volume of the aqueous dispersing phase, while varying the percentages of the dispersed phase components, to evaluate the effect of the composition on the macroscopic appearance of S-EMs (homogeneity and absence of phase separation).

As reported in Table 1, S-EM 1, S-EM 2, S-EM 5, S-EM 6, S-EM 9, and S-EM 13 appeared macroscopically homogeneous, while phase separation phenomena due to creaming of the oil phase were observed in the case of the other S-EMs.

The size distribution of S-EMs was evaluated to study the effect of composition on both droplet mean diameter, expressed as Z-Average (Z-AV), and PI values (Table 1). Z-AV of S-EMs was inversely proportional to the surfactant content. Specifically, TW80 content ≥ 7%, w/w led to Z-AV ≤ 200 nm, with the smallest droplets in the case of TW80 8%, w/w (Z-AV 13.81 nm). Conversely, TW80 ≤ 3%, w/w resulted in droplet Z-AV ≥ 400 nm.

Both EtOH and OF amounts negatively affected droplet size distribution, respectively increasing droplet diameter and PI. Specifically, EtOH ≥ 5% resulted in droplets with Z-AV > 400 nm, while OF content ≥ 4% w/w resulted in PI values ≥ 0.35, indicating an inhomogeneous size distribution. The results reported in Table 1 suggested that (i) an OF content ≥ 3% w/w hampered the production of macroscopically stable systems; (ii) an OF content 1–3% allowed to produce homogeneous S-EMs only in the presence of TW80 ≥ 7% w/w; (iii) an OF content ≤ 1%, and TW80 4% w/w led to stable and homogeneous S-EMs; (iv) TW80 ≤ 4% prevented to obtain homogeneous S-EMs.

Notably, to ensure safety for intraperitoneal administration in mice, the concentration of TW80 should not exceed 2%, as established by prior biocompatibility studies [31]. Since at this TW80 concentration, the S-EM exhibited phase separation, to address this instability without exceeding the TW80 limit, a homogenization step (Method 2) was introduced as an alternative solution, using the same S-EM compositions employed by Method (1). Nonetheless, the homogenization step did not improve the aspect of S-EMs, in some cases worsening the homogeneity, possibly due to aggregation phenomena (Table 1).

Particularly, in the case of S-EMs containing 2% TW80, despite a reduction of Z-AV, creaming phenomena were still observed (S-EM 4, S-EM 7).

Therefore, as a second attempt to stabilize the ultrastructure of S-EMs, the addition of glycerol (GL) to the composition was evaluated. Notably, considering the UroA high lipophilicity (logP 2.3), to facilitate its solubilization, the highest OF and EtOH concentrations were maintained. Namely, based on the size distribution values reported in Table 2, the composition of S-EM 7 (2% OF, 2% TW80, 6% EtOH), characterized by a mean diameter ≤ 500 nm and a PI ≤ 0.2, was taken as reference.

**TABLE 2:** Composition (%, w/w) and size distribution parameters of S-EM produced in the presence of GL, by methods (1), (2).

Formulation	OF^1^	TW80^2^	EtOH^3^	GL^4^	H_2_0	Method^5^	Z-AV^6^ (nm) ± s.d.	PI^7^ ± s.d.	Macroscopic Aspect
S-EM 16	2.0	2.0	6.0	54.0	36.0	1	862.6 ± 15.4	0.23 ± 0.01	creaming
						2	782.6 ± 20.8	0.16 ± 0.01	creaming
S-EM 17	2.0	2.0	6.0	63.0	27.0	1	801.7 ± 30.7	0.23 ± 0.03	creaming
						2	584.7 ± 22.4	0.11 ± 0.02	creaming
S-EM 18	2.0	2.0	6.0	45.0	45.0	1	760.7 ± 32.3	0.19 ± 0.04	creaming
						2	440.9 ± 24.3	0.14 ± 0.02	homogeneous
S-EM 19	2.0	2.0	6.0	81.0	9.0	1	547.2 ± 22.4	0.17 ± 0.03	creaming
						2	657.0 ± 23.8	0.16 ± 0.08	creaming

1: oil phase, blend of capric and caprylic acid triglycerides (40:60, v/v); 2: tween 80, polysorbate 80; 3: ethanol; 4: glycerol; 5: Method of S-EM preparation; 6: Z Average; 7: polydispersity index. s.d.: standard deviation; data are the mean of 4 independent determinations on different batches.

GL containing S-EMs were prepared by Methods (1) and (2), previously mixing GL with water, according to the percentages reported in Table 2.

As reported in Table 2, while the addition of GL caused a significant increase in the droplet diameters, ranging between 540 nm and 860 nm for S-EMs obtained by Method (1), the homogenization step (Method 2) enabled to reduce droplet diameter and to improve size distribution. Particularly, S-EM 18, with GL in a 1:1 weight ratio with water, underwent a 1.7-fold Z-AV decrease, and achieved a homogeneous aspect.

To further reduce the droplet size, (i) the homogenization step was prolonged from 2 to 4 min; (ii) PEG 400 (2–4%, w/w) was used both as an alternative to GL, or in addition to GL. Although both strategies led to apparently homogeneous S-EMs, with Z-AV 400–440 nm and PI 0.1-0.19, the formulations underwent phase separation phenomena after 15 days of storage.

Based on homogeneity and size distribution (Z-AV < 500 nm, and PI < 0.2), S-EM 18 prepared by Method 2, hereafter named S-EM, was selected for the solubilization of UroA (S-EM UroA).

### Preparation and characterization of UroA-loaded S-EM

UroA was loaded in S-EM at a concentration of 0.2 mg/mL. The presence of UroA did not affect the homogeneous aspect of S-EM.

Three replicates were prepared and analyzed the following day in terms of appearance, size distribution, Z-potential and pH (Supplementary Table S1).

The results of the PCS analyses revealed a homogeneous size distribution, evidenced by the low PI values. The presence of UroA did not affect the droplet size, while it increased the absolute value of the zeta potential, keeping it negative. In each case, the pH value was weakly acidic, thus compatible with intraperitoneal administration, for which a pH between 5 and 9 is appropriate [32].

To gain insights into the morphology of the produced S-EM, visualization was carried out using TEM (Supplementary Figure S1). The images evidence a disperse phase consisting of spherical droplets with diameters in agreement with PCS data.

The assessment of UroA content in the whole S-EM and of its EE in the dispersed phase was evaluated the day after preparation, by ultrafiltration, disaggregation and HPLC. As expected, the simple S-EM preparation procedure avoided possible drug degradation, resulting in a total recovery of UroA (0.2 mg/mL, 100% with respect to the weight of the drug). Notably, an almost complete UroA association within the nanodroplets was achieved (EE = 98.3%, w/w).

Both S-EM and S-EM UroA were syringeable, thus suitable for parenteral administration through a 26-G needle.

### X-ray scattering studies

**FIGURE 1:**
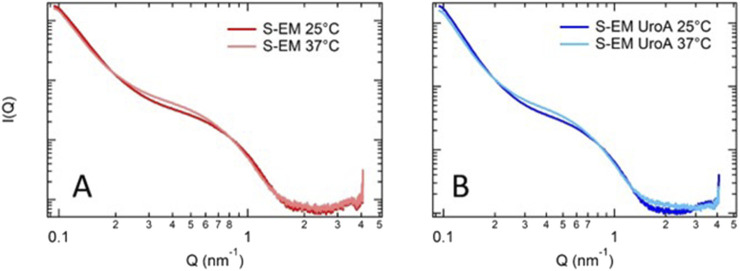
Comparison of SAXS data for S-EM **(A)** and S-EM UroA **(B)** at 25 °C and 37 °C. S-EM 18 prepared by method 2, was selected for the solubilization of UroA due to homogeneity and size distribution, thus named S-EM (S-EM UroA).

To investigate the effect of UroA on the structural organization of S-EM, and to further validate PCS results, SAXS experiments were performed. The profiles observed for S-EM and UroA-loaded S-EM are shown in Figure 1 (panels A and B, respectively). For each sample, experiments were performed at 25 and 37 °C, to mimic conditions relevant to formulation storage and systemic administration.

SAXS profiles are very similar to those reported by Cruz *et al* [33] confirming the presence of nanostructures, in which TW80 is surrounding the oil core. The absence of any peak around 0.1 nm^-1^ demonstrates the homogeneity of the internal structure of the nanodroplets.

### FT-IR characterization

**FIGURE 2:**
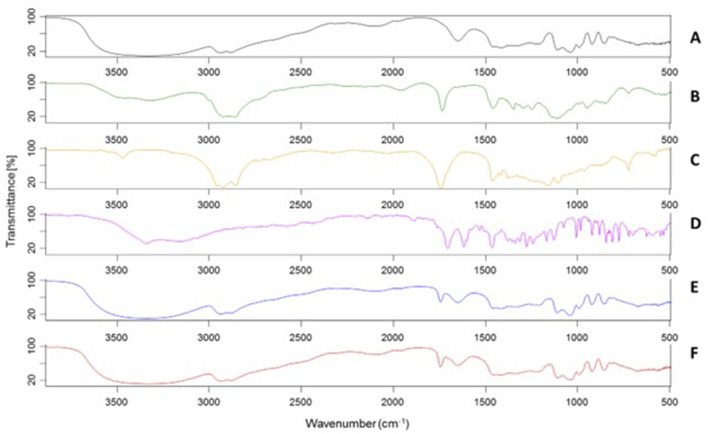
FT-IR spectra of GL **(A)**, TW80 **(B)**, OF **(C)**, UroA **(D)**, S-EM **(E)**, S-EM UroA **(F)**.

To further characterize S-EM, a study was conducted via FT-IR spectroscopy analysis. Figure 2 shows the FT-IR spectra of the excipients (A-C), UroA (D), S-EM (E), and S-EM UroA (F). It is evident that the FT-IR spectrum profiles of S-EM (E,F) are strongly influenced by the high concentration of GL (45%, w/w) in the formulation.

In the S-EM FT-IR spectrum (E), a broad band centred at 3,350 cm^−1^ is observed, characteristic of O–H stretching, along with a strong absorption at 2,935–2,881 cm^−1^ due to the stretching of aliphatic C–H bonds. A medium-intensity band at 1,745 cm^−1^, not present in GL spectrum, is typical of the C=O stretching of ester functional groups, characteristic of both OF and TW80. At 1,650 cm^−1^ the bending vibration of water is still visible, due to the natural hygroscopicity of GL. The bands at 1,110 cm^−1^ and 1,040 cm^−1^, partially overlapping, are attributable to the C–O stretching of alcoholic, ether, and ester groups present. Notwithstanding the low amount of UroA did not substantially alter the spectral profile of S-EM UroA (F), a weak shoulder at 1,706 cm^−1^ can be observed, corresponding to the intense C=O stretching band of the UroA lactone, whilst the other characteristic absorptions of the molecule are instead masked by the bands mainly attributable to GL.

### Stability studies

To evaluate the stability of S-EM UroA, PCS analyses and Z-potential evaluations were repeated 3 months after S-EM preparation. In addition, the ability of the formulation to control UroA degradation was assessed by quantifying the drug concentration over time. All formulations were stored at 4 °C and protected from light.

As reported in Supplementary Table S2, the presence of UroA did not affect the size stability of the system, as no significant changes in Z-AV and PI were recorded after 90 days. The slight variation in Z potential was compatible with the stability range for dispersed systems (≥± 25 mV). Notably, the decrease of UroA in 90 days was around 10% with respect to day one, suggesting the capability of S-EM to control UroA degradation.

### *In vitro* UroA release

**FIGURE 3:**
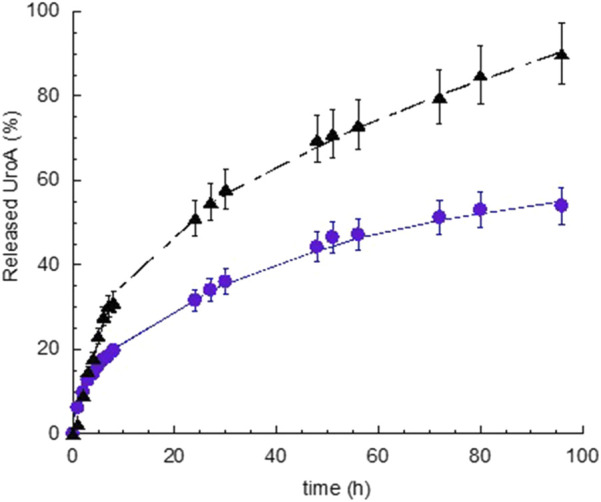
*In vitro* release kinetics of UroA from S-EM UroA (black triangle) and SUSP UroA (blue circle). UroA release represents the % with respect to UroA amount loaded in S-EM and SUSP (0.2 mg/mL). Data are expressed as the mean of 5 independent experiments ± s.d. Statistical analysis was performed using Paired t-Student test.

The release of UroA was determined *in vitro* via dialysis method, comparing S-EM UroA with SUSP UroA. As receiving phase, 0.9% NaCl and 1% TW80 was employed, maintained under constant magnetic stirring at 37 ± 1 °C. The release profiles, obtained plotted as cumulative % of drug released vs. time, are reported in Figure 3.

The release kinetics of UroA exhibited a biphasic profile, characterized by a faster release within 2 and 7 h, for S-EM UroA and SUSP UroA respectively, followed by a gradually slower drug release phase. Notably, the UroA release kinetic in the linear tract (0–7 h) was 2.35-fold faster in the case of S-EM UroA with respect to SUSP UroA, while the percentage of UroA released at 96 h was 1.7-fold higher in the case of S-EM UroA than SUSP UroA. The statistical comparison evidenced a *t*-value −5.3, and a *p-*value < 0.0001, indicating a very high statistical significance.

Zero-order plot, first-order plot, Higuchi plot and Peppas plot were applied to gain insight on the mechanism of UroA release. Supplementary Table S3 shows the R^2^ values obtained plotting the release profile according to the different mathematical models [34]. For both S-EM UroA and SUSP UroA the profile can be better described by Higuchi model, while the “n” values obtained from the Peppas plot revealed a non-Fickian diffusion mechanism. Thus, this mechanistic evaluation indicates diffusion as the primary driver. However, the Peppas model, being more sensitive to subtle changes in drug release kinetics, revealed a not purely Fickian diffusion, suggesting the involvement of additional factors that slightly modulate UroA release rate as a function of time.

### Cytotoxicity evaluation

**FIGURE 4:**
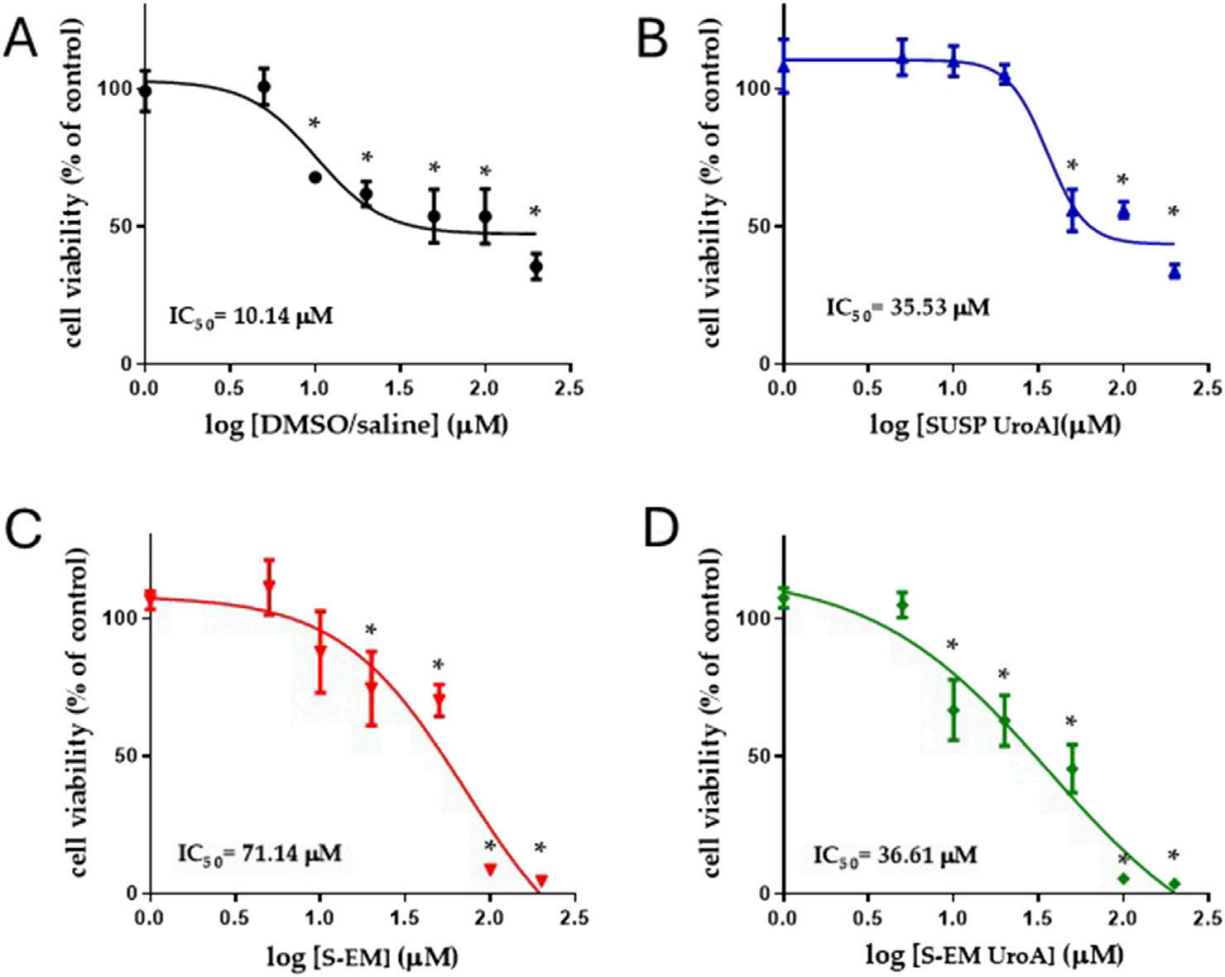
Logarithmic concentrations vs. response curves determined using MTT assay. Cell viability was evaluated by MTT assay after 48 h of treatment with DMSO/saline **(A)**, SUSP UroA **(B)**, S-EM **(C)**, and S-EM UroA **(D)**. Data represents the results of two independent experiments performed in triplicate. The IC_50_ value for each condition is shown. Results are expressed as a percentage of the control condition and are presented as mean ± s.d. Statistical analysis was performed using One-Way ANOVA followed by Dunnett’s *post hoc* test to compare differences between control and treatments; *p < 0.05.

Figure 4 shows the results of the MTT test performed on fibroblasts treated with S-EM, S-EM UroA, SUSP UroA, and DMSO/saline 1:10 (v/v) at the concentration range 1–200 μm. As expected, for all treatments the cytotoxicity increased in a concentration-dependent manner. Specifically, the most toxic concentrations were 100 and 200 µM for both S-EM (unloaded and loaded with UroA) and for the simple SUSP UroA. For concentrations up to 5 μM, S-EM UroA maintained 100% viability. Interestingly, comparing IC_50_ values of SUSP UroA and S-EM UroA, we did not observe significant differences (IC_50_ = 35.53 µM and 36.61 µM respectively), confirming that S-EM Uro A did not induce cell cytotoxicity in the concentration range 1–50 µM. This result was also confirmed by the high value of IC_50_ for fibroblasts treated with unloaded S-EM (IC_50_ = 71.14 µM).

### *In vivo* studies

**FIGURE 5:**
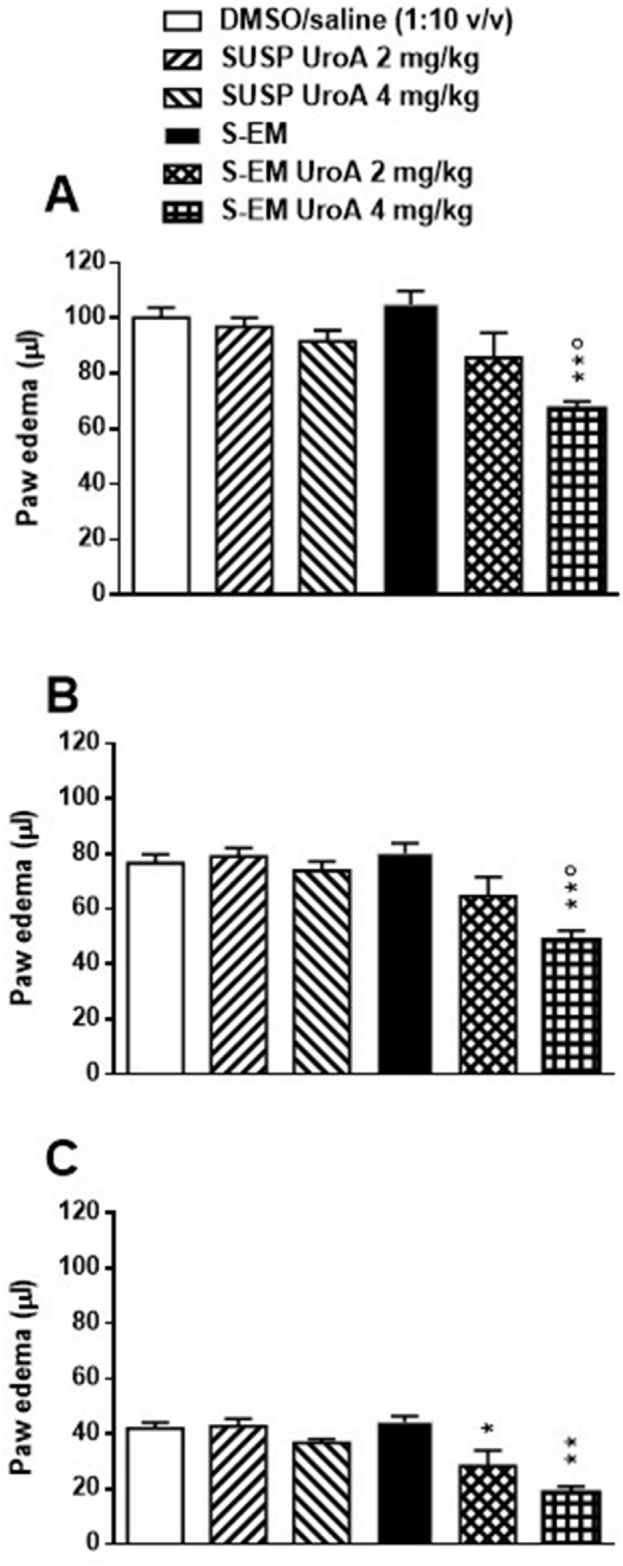
Effects of DMSO/saline (1:10 v/v), SUSP UroA 2 mg/kg, SUSP UroA 4 mg/kg, S-EM, or S-EM loaded with UroA at 2 or 4 mg/kg on carrageenan-induced paw edema in mice. Treatments were injected intraperitoneally 2 h prior to intraplantar injection of carrageenan into the hind paw. The edema was measured 3 (Panel **(A)**), 6 (Panel **(B)**) and 24 h (Panel **(C)**) after intraplantar injection and compared to the basal level. The contralateral control paw injected with saline developed no measurable edema. Mean ± SEM, n = 6. Panels **A** and **B** **p < 0.01 significantly different from DMSO/saline (1:10 v/v), SUSP UroA 2 mg/kg, SUSP UroA 4 mg/kg, and S-EM groups; °*p* < 005 significantly different from S-EM Uro A 2 mg/kg group. Panel **C** *p < 005, **p < 0.01 significantly different from DMSO/saline (1:10 v/v) and S-EM groups (one-way ANOVA followed by Tukey’s test for multiple comparisons).

As expected, intraplantar (i.pl.) injection of carrageenan induced a marked paw edema, observable at 3- and 6-h post-injection (Figures 5A,B), which moderately persisted at 24 h (Figure 5C). In contrast, the contralateral paw injected i.p. with saline showed no measurable edema.

Pretreatment with DMSO/saline (1:10 v/v), SUSP UroA 2 mg/kg, SUSP UroA 4 mg/kg or S-EM had no effect on carrageenan-induced paw edema at any time point (Figure 5). On the contrary, pretreatment with S-EM UroA at 2 mg/kg showed a trend toward edema reduction at 3 and 6 h (Figures 5A,B), with a statistically significant effect observed at 24 h post-injection (Figure 5C). Notably, S-EM UroA at 4 mg/kg significantly reduced paw edema at all measured time points, indicating a dose-dependent anti-inflammatory effect (Figure 5).

## Discussion

Recently, various studies have highlighted the efficacy of using different nanoparticle systems to deliver UroA, demonstrating improved therapeutic outcomes. For instance, Pula et al.^21^ have formulated polymeric nanoparticles and hybrid polymer-lipid nanoparticles to enhance UroA absorption in the gastrointestinal tract and mitigate cisplatin-induced kidney damage [23, 35]. Other researchers have developed solid lipid nanoparticles for targeted delivery of UroA to breast cancer cells by functionalizing them with chitosan and folic acid [36]. Multiple research groups have explored liposomes for UroA delivery, noting significant improvements in absorption and bioavailability through oral administration and demonstrating improved pharmacokinetics *in vivo* with the use of PEGylated liposomes [20]. Furthermore, a recent study evidenced the potential of UroA gelatin composite nanoparticles to inhibit tumor cell migration [37].

In this investigation S-EM were developed as UroA delivery system using a simple, low-energy consuming method, easy to scale up, based on biocompatible and regulatory-approved excipients [31]. In the design of a stable and homogeneous S-EM, surfactants are required to control of interfacial tension between the oil and water phases, providing adequate droplet stabilization, anyways excess amounts can lead to *in vivo* toxicity. The formulative study enabled to find the optimal balance between excipients, modulating OF, EtOH and TW80 concentration. Notably, GL has been shown to play a crucial role in stabilizing S-EM. Indeed, as previously reported, GL can affect the behaviour of non-ionic surfactants in water by changing their solubility and optimum curvature [38]. UroA was successfully loaded in S-EM, using a straightforward preparation method, preventing drug loss, resulting in a stable platform at least for 3 months, with physical-chemical characteristics suitable for i.p. administration of the drug. The negative Z potential, likely due to the hydroxyl functional groups of TW80, EtOH, and the OF fatty acids, was increased in its absolute value in the case of S-EM UroA, suggesting a higher stabilization. Indeed, Z potential provides information on the dispersed system stability; in particular, the electrical charge boundary constitutes a barrier at the interface between the dispersed and dispersing phases, counteracting the instability phenomena that lead to phase separation.

The SAXS data, interpreted in the low-Q Guinier region, revealed a population of droplets with an estimated diameter of ∼50 nm (e.g., R_g_ of ca. 20 nm), which likely corresponds to the lower-end of the size distribution of S-EM observed by PCS. In addition SAXS data show that S-EM structure was not affected by the drug loading. Moreover, an increase in temperature from 25 to 37 °C determined just a slight change of the scattering profile, suggesting that S-EM and S-EM UroA maintained the same structure, both after drug loading and at the administration temperature.

Notably, S-EM UroA provides improved drug solubilization and technological stability with respect to the non-physiological SUSP UroA. In this respect the faster release profile observed for S-EM UroA (2.35-fold compared to SUSP UroA) can be attributed to its efficacious solubilization within the SEM nanoproplets, making the drug more available for diffusion through the dialysis membrane towards the receiving phase. On the other hand the high lipophilicity of UroA limits its solubility in physiological media, resulting in slower release kinetics in the case of SUSP UroA. Moreover, the insolubility of UroA in the DMSO/saline (1:10, v/v) suspension led to drug sedimentation.

*In vitro* studies, performed by MTT assay on primary human fibroblasts treated with different formulations for 48 h, clearly showed the absence of toxicity at the concentrations of 5 μM and below. Although the tissue levels of UroA were not measured, it is unlikely that they could be over 5 μM at the cellular levels, when used 2 or 4 mg/kg *in vivo* [39], supporting the lack of tissue toxicity of UroA under our experimental conditions. Accordingly, it is worth noting that several studies demonstrated beneficial effects following repeated dosing regimens of UroA at substantially higher doses than those employed in this study, without reporting significant adverse effects [40].

The evaluation of the half-inhibitory concentration (IC_50_) of the different formulations, revealed that the vehicle (DMSO/saline) exhibited higher cytotoxicity than SUSP UroA (IC_50_ = 10.14 µM and 35.53 µM, respectively). This apparently contradictory result can be explained by the well-known cytotoxic effect of DMSO (at low concentrations) [18, 41, 42]. On the other hand, UroA has been demonstrated to induce an antioxidant defense mechanism [43], suggesting its ability to counteract the known prooxidative effect of DMSO [44].

*In vivo* anti-inflammatory evaluation revealed that SUSP UroA, administered at doses of 2 and 4 mg/kg, did not produce significant effects in the carrageenan-induced paw edema model. This outcome contrasts with numerous studies demonstrating the anti-inflammatory properties of UroA in various experimental systems, including macrophages, chondrocytes, and animal models of chronic inflammation [45–48]. A possible explanation for the lack of efficacy in our setting may be the relatively low doses administered and the use of a single acute treatment, whereas many previous studies employed repeated dosing regimens [40]. Furthermore, a prior study [49] reported the ability of UroA to reduce paw edema in a carrageenan model in rats; unfortunately, the specific dose used was not disclosed, making comparisons difficult. Interestingly, in that study the antinflammatory effects of UroA have been linked to its significant antioxidant properties.

Notably, when administered within the submicron emulsion (S-EM UroA), UroA at 4 mg/kg significantly attenuated inflammation at all time points, suggesting that the formulation enhances the bioactivity of the compound. At the lower dose (2 mg/kg), S-EM UroA displayed a trend toward anti-inflammatory activity at early time points and a significant effect at 24 h post-carrageenan injection. This partial efficacy is consistent with the *in vitro* release data, which showed a controlled, biphasic release of UroA from the S-EM, potentially leading to a more sustained pharmacological effect. Taken together, these findings suggest the superiority of submicron emulsions over UroA suspensions in enhancing the systemic anti-inflammatory effects of the postbiotic, likely due to an improved UroA bioavailability. It should be underlined that the pharmacological experiments presented in this study represent a preliminary step toward evaluating the *in vivo* potential advantages of S-EM compared to the UroA suspension in DMSO/saline (1:10 v/v). Further pharmacological and toxicological investigations will be necessary to confirm the efficacy of S-EM as a delivery system for UroA and to assess its ability to enhance the systemic anti-inflammatory effects of UroA without raising safety concerns. These aspects should therefore be considered as potential limitations of the present study.

## Conclusion

In conclusion, a S-EM constituted of excipients compatible with parenteral administration was successfully developed and characterized. We demonstrated its ability to effectively solubilize the lipophilic UroA, without significantly altering the mean droplet diameter, which consistently remained ≤ 500 nm, suitable for intraperitoneal administration. SAXS technique demonstrated that S-EMs maintained their ultrastructure when loaded with the drug. Furthermore, S-EM UroA proved to be macroscopically and dimensionally stable for at least 90 days. *In vitro* release studies evidenced a faster UroA release in the case of S-EM Uro A, with respect to SUSP UroA, due to the higher capability of S-EM to solubilize the drug.

*In vivo* studies conducted in mice via intraperitoneal administration demonstrated the potential of S-EM UroA as an effective delivery system for UroA. It showed superiority over simple SUSP UroA in enhancing the systemic anti-inflammatory effects of the postbiotic, possibly through enhanced UroA bioavailability.

These results highlight the efficacy of S-EM as a delivery system for UroA, demonstrating its capability to outperform simple suspensions in enhancing the drug systemic anti-inflammatory effects. Therefore, this formulation may represent a suitable delivery system for future studies investigating the therapeutic effects of UroA, also administered via alternative routes (*i.e.*, oral and intranasal), in the context of neurodegenerative diseases. From this perspective, further research is warranted to assess the ability of S-EM UroA to cross the blood-brain barrier, to evaluate its safety profile in neuronal cells, and to determine its therapeutic efficacy in relevant animal models, thereby providing a comprehensive framework for the potential clinical translation of this innovative formulation.

## Data Availability

The original contributions presented in the study are included in the article/Supplementary Material, further inquiries can be directed to the corresponding authors.
