# Supplementary material for: Development of a submicron emulsion-based delivery system to improve the anti-inflammatory activity of urolithin A
Source: J Pharm Pharm Sci. 2025 Nov 7;28:15553. doi: 10.3389/jpps.2025.15553 (PMC12634460; doi:10.3389/jpps.2025.15553)
Supplement: Supplementary file 1 [file Supplementaryfile1.docx]

**SUPPLEMENTARY MATERIALS**

**Table 1S.** Z-Average, Polydispersity index, pH and Z-potential of indicated formulations.

| Formulation | Z-AV^1^  (nm) ± s.d. | PI^2^  ± s.d. | pH  ± s.d. | Z-potential  ± s.d. |
| --- | --- | --- | --- | --- |
| S-EM | 440.9 ± 24.3 | 0.14 ± 0.02 | 5.74 ± 0.05 | -15.92 ± 0.81 |
| S-EM UroA | 440.3 ± 53.6 | 0.12 ± 0.03 | 5.77 ± 0.10 | -22.99 ± 9.83 |

1: Z Average; 2: polydispersity index. s.d.: standard deviation; data are the mean of 3 independent determinations on different batches. S-EM corresponds to S-EM 18 prepared by method 2, accordingly, S-EM UroA is S-EM 18 prepared by method 2 loaded with UroA0.2 mg/ml.

**Table 2S.** Variation of size, Zeta-potential and EE of S-EM UroA stored for 3 months at 4 °C.

| Time (days) | Z-AV^1^  (nm) ± s.d. | PI^2^  ± s.d. | Z-potential  ± s.d. | UroA EE^3^ |
| --- | --- | --- | --- | --- |
| 1 | 440.3 ± 53.6 | 0.12 ± 0.03 | -22.99 ± 9.83 | 100.0 ± 0.9 |
| 30 | 420.5 ± 44.3 | 0.13 ± 0.01 | -23.35 ± 5.40 | 99.5 ± 0.2 |
| 60 | 414.1 ± 36.8 | 0.14 ± 0.09 | -26.45 ± 5.32 | 94.0 ± 0.1 |
| 90 | 407.5 ± 79.6 | 0.16 ± 0.04 | -28.27 ± 3.44 | 90.5 ± 0.5 |

1: Z Average; 2: polydispersity index; EE: entrapment efficiency; s.d.: standard deviation; data are the mean of 4 independent determinations on different batches. S-EM corresponds to S-EM 18 prepared by method 2, accordingly, S-EM UroA is S-EM 18 prepared by method 2 loaded with UroA 0.2 mg/ml.

**Table 3S.** IVRT parameters and kinetic data of the indicated formulations.

| Formulation | R¹  (µg/cm^2^/h) | A^2^  (µg/cm^2^) | Zero Order  Plot (R^2^) | First Order  Plot (R^2^) | Higuchi  Plot (R^2^) | Peppas  Plot (n/R^2^) |
| --- | --- | --- | --- | --- | --- | --- |
| S-EM UroA | 4.63 ± 0.95 | 96.01 ± 14.23 | 0.987 | 0.994 | 0.996 | 1.27/0.963 |
| SUSP UroA | 1.97 ± 0.22 | 54.11 ± 7.32 | 0.955 | 0.963 | 0.992 | 0.56/0.991 |

1: Release rate; 2: amount of UroA released after 96 h; UroA concentration was 0.2 mg/mL; data are the mean of 5 independent Franz cell experiments ± s.d. S-EM corresponds to S-EM 18 prepared by method 2, accordingly, S-EM UroA is S-EM 18 prepared by method 2 loaded with UroA 0.2 mg/ml. SUSP UroA corresponds to a DMSO/saline 1:10, (v/v),suspension loaded with Uro A 0.2 mg/ml.


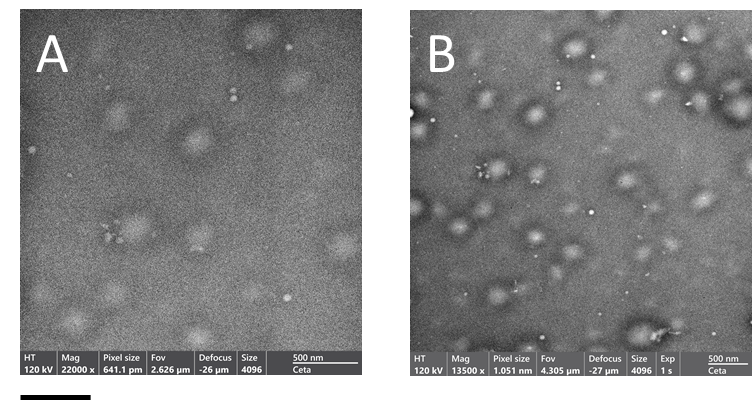


**Figure 1S.** TEM images of S-EM (A) and S-EM UroA (B). Bar corresponds to 500 nm and 700 nm in panels A and B respectively. S-EM corresponds to S-EM 18 prepared by method 2, accordingly, S-EM UroA refers to S-EM 18 prepared by method 2 loaded with UroA.
